# Ti_3_C_2_T_x_ MXene-Coated Electrospun PCL Conduits for Enhancing Neurite Regeneration and Angiogenesis

**DOI:** 10.3389/fbioe.2022.850650

**Published:** 2022-03-16

**Authors:** Li-Ping Nan, Zeng Lin, Feng Wang, Xue-Han Jin, Jia-Qi Fang, Bo Xu, Shu-Hao Liu, Fan Zhang, Zhong Wu, Zi-Fei Zhou, Feng Chen, Wen-Tao Cao, Jian-Guang Wang, Jun-Jian Liu

**Affiliations:** ^1^ Department of Orthopedic, Shanghai Tenth People’s Hospital, School of Medicine, Tongji University, Shanghai, China; ^2^ Department of Spine Surgery, Shanghai East Hospital, School of Medicine, Tongji University, Shanghai, China

**Keywords:** MXene, electrical stimulation, electrospinning, peripheral nerve injuries, vascular endothelial growth factor, nerve guide conduits

## Abstract

An electrical signal is the key basis of normal physiological function of the nerve, and the stimulation of the electric signal also plays a very special role in the repair process of nerve injury. Electric stimulation is shown to be effective in promoting axonal regeneration and myelination, thereby promoting nerve injury repair. At present, it is considered that electric conduction recovery is a key aspect of regeneration and repair of long nerve defects. Conductive neural scaffolds have attracted more and more attention due to their similar electrical properties and good biocompatibility with normal nerves. Herein, PCL and MXene-PCL nerve guidance conduits (NGCs) were prepared; their effect on nerve regeneration was evaluated *in vitro* and *in vivo*. The results show that the NGCs have good biocompatibility *in vitro*. Furthermore, a sciatic nerve defect model (15 mm) of SD rats was made, and then the fabricated NGCs were implanted. MXene-PCL NGCs show similar results with the autograft in the sciatic function index, electrophysiological examination, angiogenesis, and morphological nerve regeneration. It is possible that the conductive MXene-PCL NGC could transmit physiological neural electric signals, induce angiogenesis, and stimulate nerve regeneration. This paper presents a novel design of MXene-PCL NGC that could transmit self-originated electric stimulation. In the future, it can be combined with other features to promote nerve regeneration.

## Introduction

Peripheral nerve injury (PNI) can lead to persistent neurological damage in children and adults due to motor vehicle accidents, combat trauma, neoplasms, and vascular and compression disorders (Foster et al., 2018; Karsy et al., 2019; Dervan et al., 2021). Inadequate functional outcomes as nerves slowly regenerate is deemed a fundamental challenge of successful PNI recovery (Black and Lasek, 1979). At present, although autologous nerve transplantation is still the gold standard for the treatment of PNI, a lack of donor nerve sources and dysfunction of the donor site are the main limitations in clinical use (Liu et al., 2022; Wang et al., 2022). Nerve guidance conduits (NGCs) can provide a specific microenvironment for nerve repair and regeneration, promote peripheral nerve regeneration through various approaches, and become a potential substitute for autologous nerve, which has important research value and clinical application prospects (Qian et al., 2018b; Fregnan et al., 2020; Yoo et al., 2020; Zhang et al., 2020; Qian et al., 2021a; Qian et al., 2021b).

Biodegradable materials, such as polycaprolactone, chitosan, and polylactic acid, are used to fabricate NGCs in many studies (Barroca et al., 2018; Qian et al., 2020; Sun et al., 2021; Zeng et al., 2021). Electrical signals play an important role in normal nerve function and nerve regeneration (Qian et al., 2019; Qian et al., 2020; Wang et al., 2020; Kasper et al., 2021). The conductive NGC is a promising substitute for autograft in repairing PNI (Zhang et al., 2020; Zhao et al., 2020; Jin et al., 2021; Song et al., 2021). Generally, a perfect NGC usually needs to afford the appropriate mechanical properties to provide a cavity for nerve regeneration and good electrical conductivity to transmit nerve signals, promote nerve regeneration and myelination, and prevent scar tissue infiltration (Sun et al., 2019; Yan et al., 2020; Senger et al., 2021). The latest studies indicate that the electrophysiological properties of the biomaterial could be improved after being blended in the conductive particles or polymers, such as graphene, carbon nanotubes (CNTs), and Ti_3_C_2_T_x_ MXene (Qian et al., 2018a; Mao et al., 2020; Rastin et al., 2020; Zhang et al., 2021). The cytotoxicity of CNTs limits their usage in the clinic (Andón and Fadeel, 2013; Zhang et al., 2022). Similarly, graphene could also lead to cytotoxicity due to its dosage (Shin et al., 2016). On the contrary, Ti_3_C_2_T_x_ MXene has gradually become a research hot spot in the field of electronic biosensing and tissue engineering due to its excellent characteristics, such as decent biocompatibility, large specific surface area, and good electrical conductivity and hydrophilicity (Xu et al., 2016; Cao W. T. et al., 2019; Basara et al., 2020; Wang et al., 2021; Wei et al., 2021; Zhou et al., 2021). Ti_3_C_2_T_x_ MXene can promote proliferation and differentiation in BMSCs, transmit electric signals, and accelerate tissue regeneration (Huang et al., 2020; Mao et al., 2020). We believe that the MXene composite nanofibers could be an ideal choice to provide a good microenvironment for nerve regeneration considering its excellent biocompatibility and unique nanofibrous structure. This study is aimed to explore the effects of MXene on nerve regeneration in PNI. Here, we engineer a MXene-PCL NGC by using electrospinning and spray-coated technology. The obtained MXene-PCL conduit could afford a good environment for RSC adhesion and proliferation on the PCL side. The MXene-PCL shows excellent performance in promoting nerve regeneration in a long-range sciatic nerve defect model in Sprague Dawley (SD) rats. We found that the MXene-PCL NGCs showed similar results with the autograft in restoring the nerve structure and function. These data suggest that MXene plays an important role in neural regeneration.

## Materials and Methods

### Materials

The PCL pellets (CAPA6800, Perstorp Ltd, Sweden, Mw = 80,000 kDa) used in this experiment are medical grade (approved by the United States Food and Drug Administration), and melt at 60°C. Lithium fluoride (LiF ≥99%) and 2,2,2-trifluoroethanol (TFE) were purchased from Sigma (St. Louis, MO). Hydrochloric acid (HCl), absolute ethyl alcohol, and absolute methyl alcohol were purchased from Aladdin Reagents (Shanghai) Co., Ltd., China. The Ti_3_AlC_2_ powder was purchased from Jilin 11 technology Co., Ltd. All reagents were analytical grade.

### Fabrication of Delaminated Ti_3_C_2_T_x_ (D-Ti_3_C_2_T_x_) MXene

The d-Ti_3_C_2_T_x_ was synthesized by a typical chemical exfoliation process as we described previously with some modifications (Cao W.-T. et al., 2019). Typically, 1 g of Ti_3_AlC_2_ powder was dissolved in a mixture of 9 M HCl (20 ml) and 1 g LiF. Then, the obtained suspension was allowed to process for 48 h at 35°C in a water bath for etching the aluminum (Al) layer. The resultant suspension was washed repeatedly using deionized water until its pH reached about 5. Finally, the obtained sediment was further delaminated by ultrasonication in an ice bath and centrifuged to obtain uniform d-Ti_3_C_2_ MXene nanosheets suspension. The obtained MXene nanosheets suspension was further stored in a refrigerator at 4°C to prevent oxidation.

### Fabrication of PCL and MXene−PCL NGCs

For the preparation of pure PCL NGCs, 2 g of PCL pellets was added into 12 ml of trifluoroethanol to form 17% (w/v) transparent adhesive solution with stirring for 24 h at room temperature. The NGCs were prepared by using electrospinning equipment (Changsha Nayi Instrument Technology Co., Ltd, China). Briefly, the obtained solutions were transferred into a 5-ml syringe and electrospun at 0.8 ml/h under 12-kV high DC voltage. The distance from the rotational collector (200 rpm, a tungsten steel rod with a diameter of 2 mm) to the metal needle is 25 cm. Then, the obtained PCL NGCs were gently detached. To prepare MXene-PCL NGCs, MXene solution (an aqueous solution containing 40 mg ml−^1^ MXene) was uniformly coated on the outer surface of PCL NGC by spraying technology, and the thin conductive sheath was formed by spontaneous adhesion. In addition, we tried to coat the MXene on a variety of other materials (bacterial cellulose membrane, textiles, paper, Latex, and melt-blown fabric).

### Characterization of the NGCs

The morphology and surface structure of MXene-PCL and PCL NGCs were characterized by a digital camera and scanning electron microscope separately, and the size and morphology of MXene nanosheets were examined using a transmission electron microscope (TEM, JEOL JEM-2010 (HT)). Fourier-transform infrared (FTIR) spectroscopy spectra was obtained using a FTIR spectrometer (Nicolet iS 10). The conductivity capability of the nano scaffolds was measured using four-point probes (ST2258C, China). The tensile stress–strain mechanical property was evaluated using an electronic tensile test machine (HY-940FS, Shanghai, China). To measure the porosity (%) of PCL scaffolds, we obtained a certain volume of PCL scaffolds and weighed the mass to calculate the density ρ_1_. Furthermore, the density of PCL particles is measured as ρ_2_. Then the porosity (%) of the PCL scaffolds = (1 − (ρ_1_/ρ_2_)) × 100%. Similarly, the density of MXene-PCL NGC was measured as ρ_3_, the bulk density of MXene and PCL was calculated as ρ_4_, and the porosity (%) of the MXene-PCL scaffolds = (1 − (ρ_3_/ρ_4_)) × 100%.

### Cell Viability Assays

Rat Schwann cells (RSCs) were provided by the Chinese Academy of Sciences (Shanghai, China). The MXene-PCL and PCL film were soaked in 75% alcohol and rinsed repeatedly with PBS at least three times. Then, they were placed in a sterile Petri dish, dried in a fume hood, and irradiated on the front and back sides for 2 h separately to achieve sterilization by an ultraviolet lamp. We seeded the cells on the PCL side of MXene-PCL scaffolds, PCL scaffolds, and TCP at a density of 2 × 10^4^ cm^−2^. After incubation for 24 h, a LIVE/DEAD kit (Beyotime, China) was used for cell viability analysis according to the standard protocols. Finally, cells in each group were photographed under a fluorescence microscope.

### Cytotoxicity Assay

The Cell Counting Kit 8 (CCK-8, Beyotime, China) was used to assess cytotoxicity. The cells were cultured on the PCL side of MXene-PCL scaffolds and PCL scaffolds in 24-well plates for 6, 12, 24, 72, and 168 h. Subsequently, a CCK-8 detection solution was added to each well to ensure that the concentration of CCK-8 in the medium solution was 10%. After incubation at 37°C for 3 h, the absorbance of the supernatant was detected by a microplate reader (Thermo 3,001, Thermo Fischer Scientific, United States) at a wavelength of 450 nm. TCP was used as a control. This was repeated at least three times for each group.

### Cell Morphology

RSCs were seeded onto the PCL side of MXene-PCL nanofiber membrane, and PCL nano scaffolds separately. After 4 days, the morphology of the cells on the different scaffolds were observed by scanning electron microscopy (ZEISS Gemini 300). First, the medium was discarded, washed with PBS, and fixed with 2.5% glutaraldehyde at room temperature for 2 h. We added 1% osmium acid and incubated at 4°C for 2 h after discarding the fixation solution, followed by alcohol gradient (10, 30, 50, 70, 80, 90, 95, 100%) dehydration for 15 min each. After drying at room temperature, SEM pictures were taken to evaluate the adhesion and morphology of cells on the different scaffolds.

In addition, phalloidin staining was also used to observe the adhesion ability and morphology of RSCs on the different scaffolds. After the cells were cultured on MXene-PCL and PCL nanoscaffolds for 4 days, the cell culture medium was discarded and washed with PBS twice. Then, the cells were fixed in 3.7% formaldehyde solution prepared by PBS at room temperature for about 20 min and washed with PBS containing 0.1% Triton X-100 three times, 5 min each. Actin-tracker Red (Beyotime, China) was diluted with PBS containing 5% BSA (Beyotime, China) and 0.1% Triton X-100 (Beyotime, China) at a ratio of 1:200. The solution was added to each slide in a ratio of 200 µl and incubated in a slide dyeing box at room temperature away from light for 60 min. Then, we washed the cells with PBS containing 0.1% Triton X-100 three times, 5 min each. Finally, a fluorescence microscope was used for observation.

### *In Vitro* Degradation

The NGCs were dried to a constant weight, and the sample mass W_0_ was recorded. Then, we placed the samples in a quantitative PBS solution at 37°C. At fixed time points, each sample was dried and weighed W_1_. The formula of mass loss rate W% is as follows: W% =(W_0_−W_1_)/W_0_×100%.

### Animal Surgery

SD rats (male, weighing 150–200 g) were used to evaluate the nerve regeneration capacity. The modal rats were assigned into three groups randomly: PCL, autograft, and MXene-PCL groups. Animals were deeply anesthetized with 2% pentobarbital sodium. The sciatic nerve and its main branches were exposed, and a 15-mm long nerve defect was created under aseptic conditions. In the autograft group, the nerve was sutured end to end after rotating the nerve stump around 180°. In the PCL or MXene-PCL groups, the nerve defect was sutured with a nerve conduit. After that, the soft tissue and skin were sequentially sutured. To prevent infection, antibiotic (ceftriaxone sodium, 0.1 g/kg) was injected for three consecutive days. Postoperative assessments took place at weeks 4, 8, and 12. Ethical approval for animal studies was obtained from the Institutional Animal Care and Use Committee of Shanghai Tenth People’s Hospital (SHDSYY-2021–2,305).

### Sciatic Nerve Electrophysiological and Function Analysis

The rats’ footprints were acquired to calculate the sciatic functional index (SFI) at weeks 4, 8, and 12 postoperative. The formula used to measure nerve function was as follows: SFI = (13.3 × (EIT − NIT)/NIT) + (−38.3 × (EPL − NPL)/NPL) + (109.5 × (ETS − NTS)/NTS) − 8.8. In this formula, the IT stands for the distance from the second to the fourth toes; the PL represents the length from the distal of the third toe to the heel; TS is the length from the first to the fifth toe, N means the normal side, and E means the experimental side. The SFI is scored from 0 (normal function) to 100 (the function is completely impaired). Electrical signals such as compound motor action potential (CMAP) and nerve conduction velocity (NCV) were recorded by electrophysiology analysis at 12 weeks postoperative.

### Gastrocnemius Muscle Weight Analysis

The wet weight of the gastrocnemius muscle was measured at week 12 after implantation. The gastrocnemius muscles from both legs were dissected, and the fat tissues attached were carefully removed with ophthalmic micro-scissors. The normalized wet weight of gastrocnemius muscle (%) = Wo/Wn, where Wo is the gastrocnemius muscle weight of the operated leg and Wn is the gastrocnemius muscle weight of the nonoperated leg.

### Histological Analysis

Regenerated nerves were collected on completion of the electrophysiological experiments at week 12 postoperatively. The cross-sectional morphologies of the nerves (in the middle of the specimens) were visualized by TB staining, hematoxylin-eosin (HE) staining, and TEM. For HE staining, the samples were fixed with 4% paraformaldehyde and then embedded in paraffin, cut into sections by using a microtome. For TEM and TB staining, the tissues were fixed at 4°C in 2.5% glutaraldehyde, followed by embedding in Epon812, cut into ultrathin sections. The sections were observed using an immunofluorescence microscope (Leica, United States ). The superfine microstructure of the regenerated myelin sheath was observed via a TEM (China Titan) at a voltage of 80 kV. The diameter and density of the axon and the thickness of the myelin sheath were quantitatively analyzed by ImageJ software. The S100/MBP and NF200/Tuj1 triple immunofluorescence staining were used to assess nerve myelin protein and nerve axon protein of the regenerated nerve, respectively. Tissues were fixed with 1% tetraoxide solution, dehydrated, and embedded in Epon812 resin. The cross-section was cut to a thickness of 4 μm and mounted on 2% gelatin-coated slides. The primary antibodies (Abcam, United States) included anti-Tuj1 (1:200), anti-MBP (1:200), anti-NF 200 (1:200), and anti-S100 (1:200). All slides were evaluated using an immunofluorescence microscope (Leica, United States). At week 12 after surgery, the paraffin sections of regenerated sciatic nerve sections were prepared, respectively, for immunofluorescence staining with CD34 antibody and immunohistochemistry (ICH) staining with CD31 antibody as described above. The primary antibodies included rabbit anti-CD34 antibody (1:200, ABclonal, A7429) and rabbit anti-CD31 antibody (1:200, Bioss, bs-0195R). The microvessel density (MVD) and CD 31 areas were measured by ImageJ software. The morphology of muscle fibers (from the gastrocnemius muscle of the operated leg) and major organs (heart, liver, spleen, lung, and kidney) were evaluated by HE staining.

### Statistical Analysis

Images from immunofluorescence staining, TEM, HE, and immunohistochemistry staining were analyzed using Prism 8 (GraphPad, United States) and ImageJ software. All measurements were performed five times, and the results are presented as the mean ± s.d. Differences between the values were analyzed with one-way analysis with Tukey’s *post hoc* with GraphPad Prism. For all figures, NS: *p* > *.05*; *p < *.05*.

## Results

### Fabrication and Characterization of PCL and MXene-PCL NGCs

**FIGURE 1:**
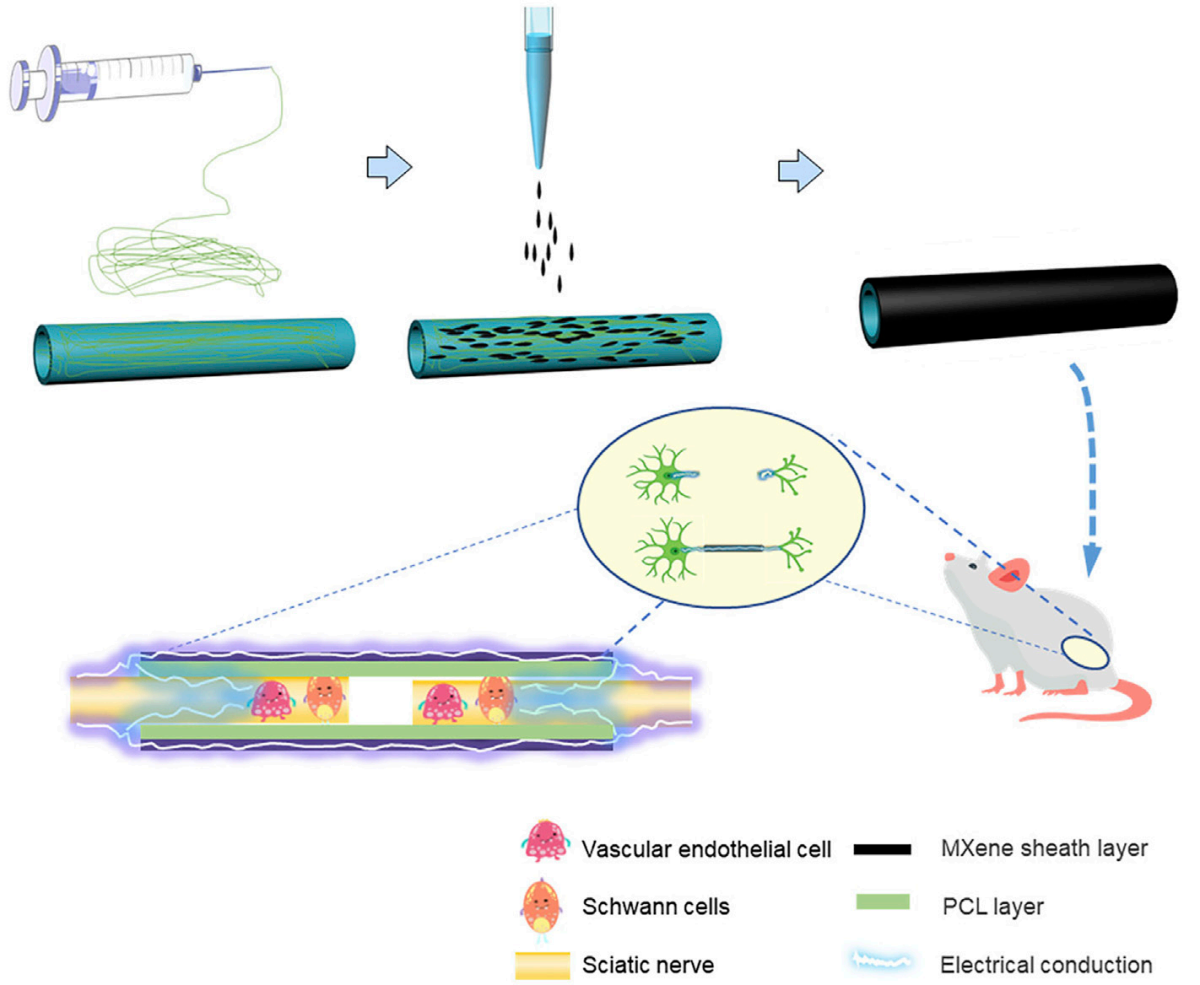
Schematic summary illustration of the work conducted in this article. The PCL NGCs were prepared by electrospinning, to prepare MXene-PCL NGCs, MXene solution was uniformly coated on the outer surface of PCL NGC by spraying technology. The obtained MXene-PCL NGCs that could transmit self-originated electrical stimulation were implanted in the rat model and finally repaired the peripheral nerve defects.

**FIGURE 2:**
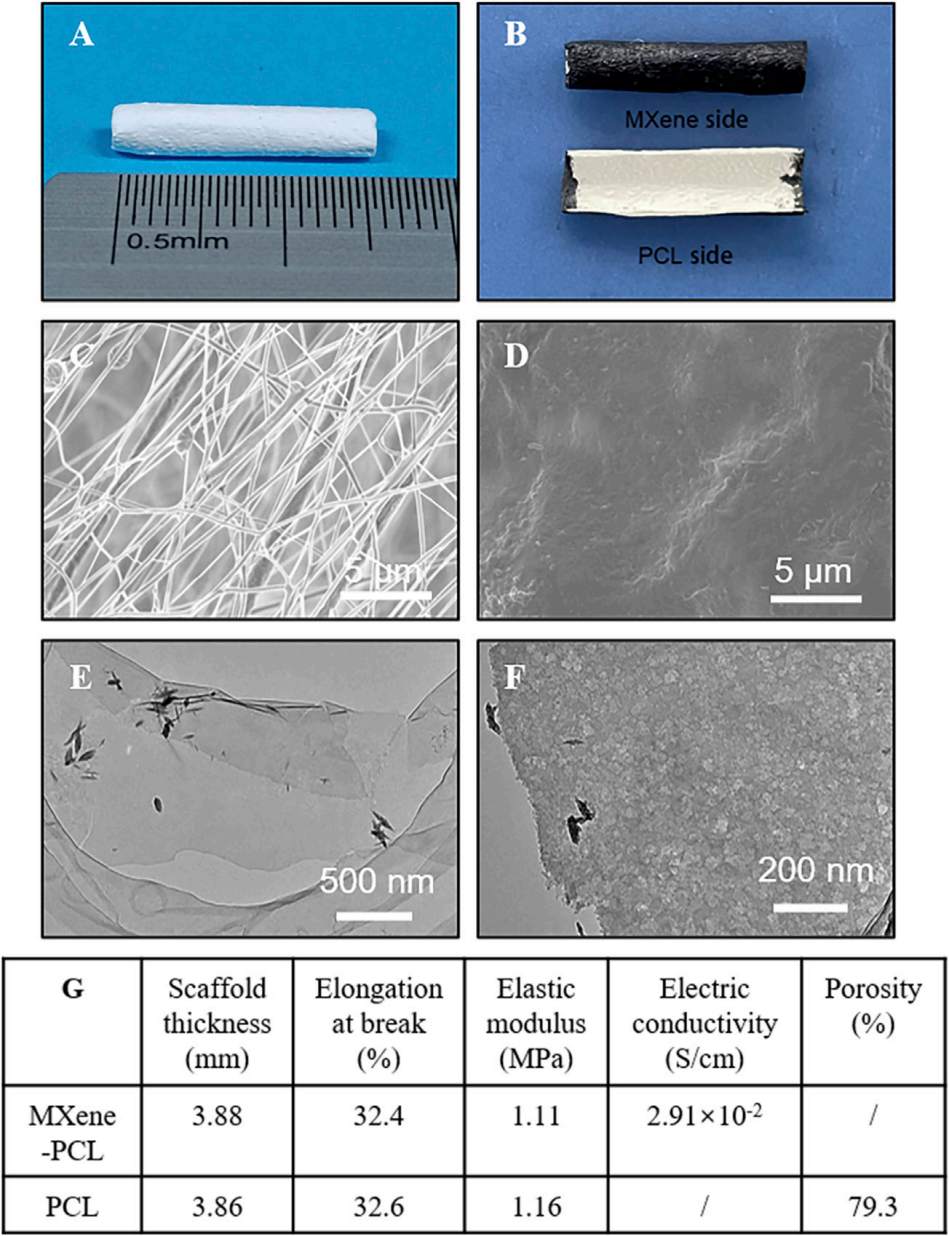
Materials characterization. Representative optical microscope pictures of the PCL NGC **(A)**, and MXene-PCL NGC **(B)**. SEM images photographed from PCL side (inner surface) **(C)** and the Mxene side (outer surface) of the MXene-PCL NGC. Low- **(E)** and high-magnification TEM image **(F)** of d-Ti_3_C_2_ MXene nanosheets. Summarizing table of the MXene-PCL and PCL scaffolds’ mechanical and electrical characteristics **(G)**.

Long gap nerve defect still is a common clinical disease with poor prognosis, and the conductive NGCs may have clinical utility in curing this disease. MXene shows good electrical conductivity, could promote tissue regeneration, and has potential application in neural tissue engineering. There is extensive literature supporting that the optimal porosity of the NGC is a critical feature for achieving neovascularization, modulating the immune response, and nerve growth patency (Shen et al., 2021; Yu et al., 2021). In this study, we fabricated PCL NGCs by electrospinning (Figure 1), which had a porous structure and appropriate mechanical properties, and size (Figure 2). To prepare MXene-PCL NGCs, MXene solution was uniformly coated on the outer surface of PCL NGC by spraying technology, and the thin conductive sheath was formed by spontaneous adhesion. Thus, the MXene-PCL NGC with PCL inner cavity and MXene outside sheath was obtained finally. The morphologies of MXene-PCL and PCL NGCs were first characterized using an optical microscope (OM) and SEM (Figure 2). The obtained PCL NGC has a milky white color, and the MXene-PCL NGC showed a white inner cavity and a black shell. The infrared absorption peak of MXene-PCL composite film (MXene side as the front side) was completely consistent with those of pure MXene film, indicating that MXene formed dense films on the surface of PCL through self-adhesion (Supplementary Figure S1, Supporting Information). Further tests indicated that the MXene is also easy to coat on other materials (including bacterial cellulose membrane, textiles, paper, latex, and melt-blown fabric) (Supplementary Figure S2, Supporting Information). SEM images obtained from the PCL side of MXene-PCL film showed a loose porous appearance formed by PCL fibers interlaced. Meanwhile, the MXene sheath is relatively smooth. TEM analysis further revealed the MXene had a lamellar nanostructure. We further estimated the mechanical and electrical properties of the MXene-PCL and PCL NGCs. The scaffold thickness, average elastic modulus, and the elongation at break, and porosity results were similar for both materials (Figure 2 and Supplementary Figure S3, Supporting Information). Therefore, the addition of the MXene sheath is too thin to influence these properties. The electrical conductivity changed from 0 S/cm (PCL NGC) to 2.91 × 10^–2^ S/cm (MXene-PCL NGC) after coating of MXene sheath. According to the results presented above, the MXene-PCL NGC exhibited excellent mechanical and topological properties, ideal rigidity and flexibility, microporosity for cell adhesion and proliferation in the PCL side, and relatively high electrical conductivity on the outer surface.

### Biocompatibility of the NGCs

**FIGURE 3:**
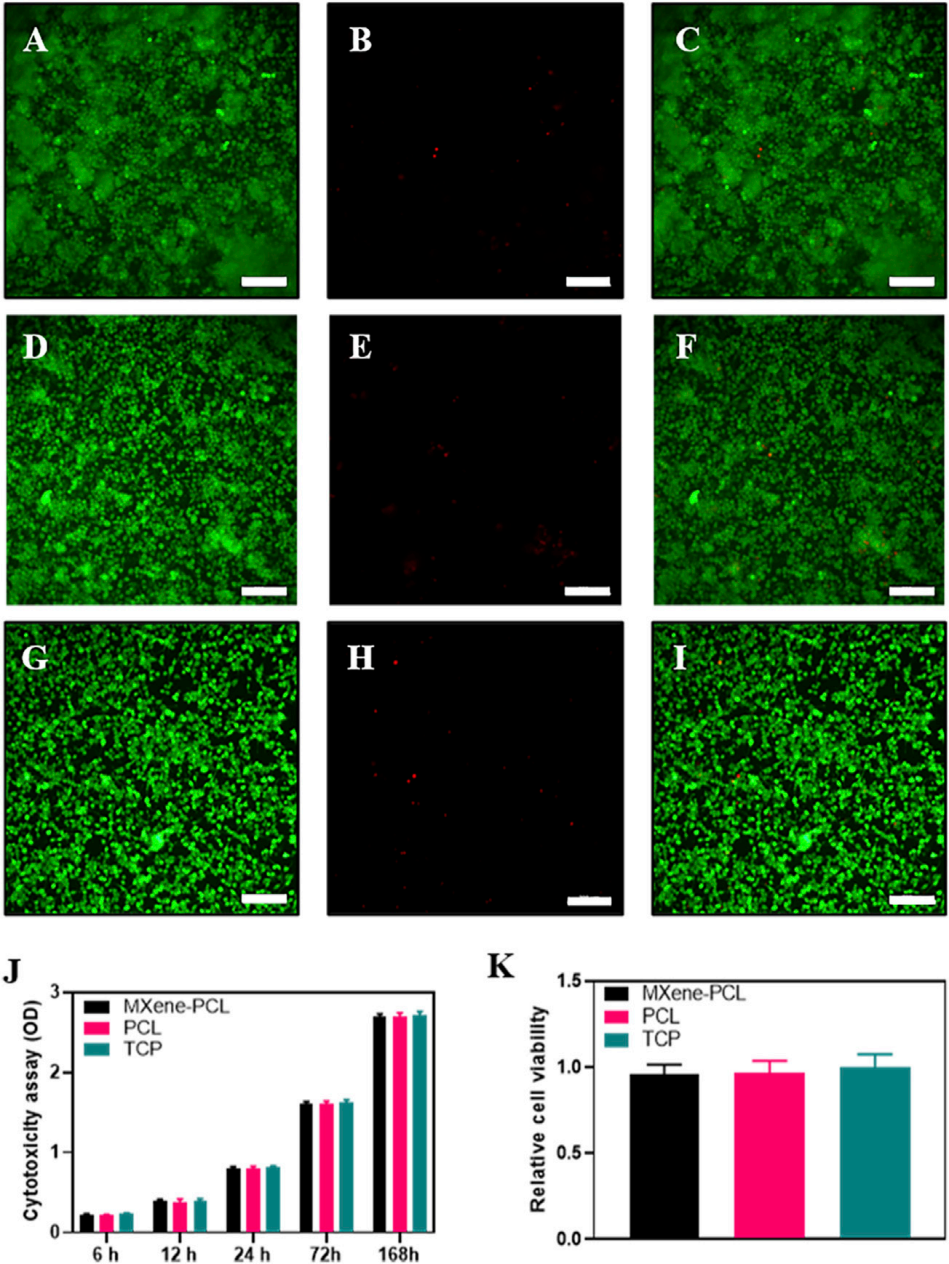
Biocompatibility assessments. LIVE/DEAD cell staining on MXene-PCL scaffolds **(A–C)**, PCL scaffolds **(D–F)** and TCP **(G–I)**. Live cells (green fluorescence, **(A,D,G)**. Dead cells (red fluorescence, **(B,E,H)**. Merged images **(C,F,I)**. Cytotoxicity was monitored by CCK-8 assay for RSCs cultured on MXene-PCL scaffolds, PCL scaffolds, and TCP at 6, 12, 24, 72, and 168 h J). Relative cell viability was evaluated by the LIVE/DEAD cell staining for MXene-PCL scaffolds, PCL scaffolds and TCP **(H)**. Experiments were repeated three times. The scale bar is 200 μm.

**FIGURE 4:**
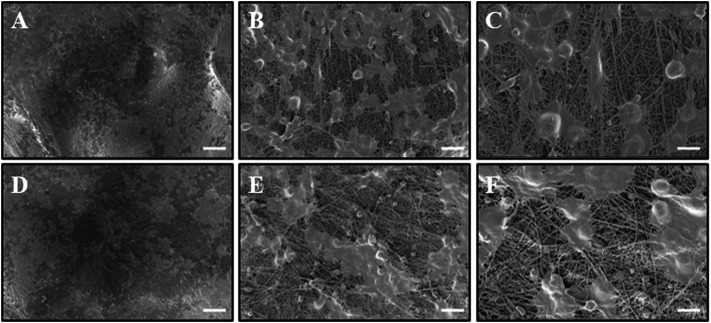
Observation of RSC morphology on the PCL side of MXene-PCL and PCL NGC scaffolds through SEM. RSCs were seeded on the MXene side of MXene-PCL scaffold **(A–C)** and PCL scaffold **(D–F)** for 72 h before being examined by SEM. The scale bars are 100 μm **(A,D)**, 20 μm **(B,E)**, and 10 μm **(C,F)**, respectively.

**FIGURE 5:**
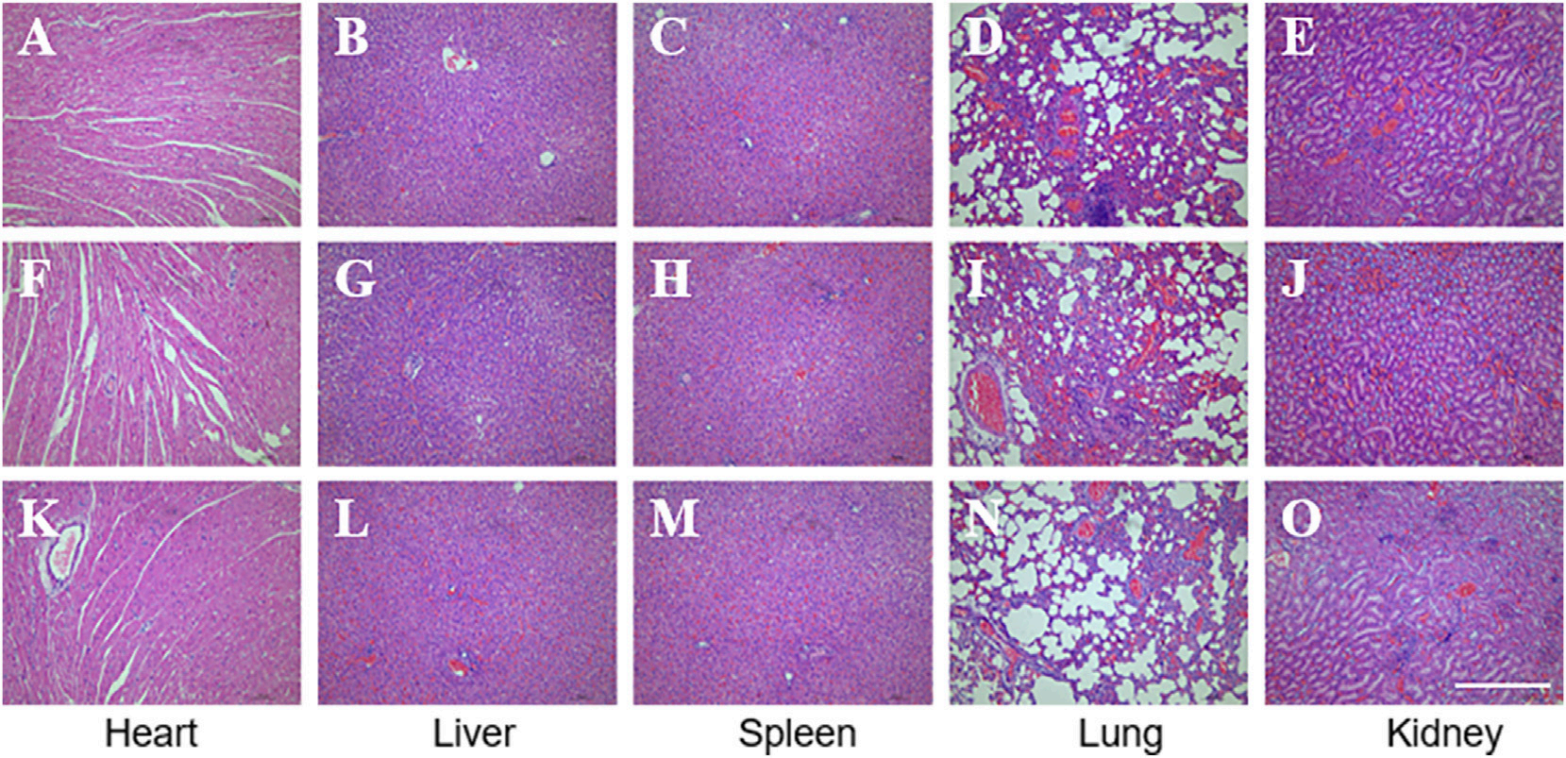
Biosafety evaluation of MXene-based nano scaffolds *in vivo*. HE staining of the major functioning organs of Mxene-PCL group **(A–E)**, PCL group **(F–J)**, and autologous nerve graft group **(K–O)**. The scale bar is 400 μm.

Biosafety is always a considerable concern for potential clinical applications of electroactive nanoparticles, such as MXene. Previous research claims that MXene-contained materials were noncytotoxic, healable, and degradable (Xu et al., 2016; Wang et al., 2019; Basara et al., 2020; Wang et al., 2021; Wei et al., 2021; Zhou et al., 2021). The design in which the MXene was coated outside the conduit rather than inside could avoid the potential toxicity caused by direct contact between the MXene particles and newly regenerated nerves to some extent. For this study, we used Schwann cells to evaluate the potential toxicity of PCL or MXene-PCL scaffold at different time points. According to CCK-8 results, Schwann cells on both scaffolds were active and showed no statistically significant differences in cell proliferation after culturing for 6, 12, 24, 72, and 168 h compared with the TCP controls (Figure 3). The LIVE/DEAD test results revealed negligible differences among the TCP, MXene-PCL, and TCP groups (Figure 3). Cell attachment on the scaffolds is a crucial factor for cell viability. The morphology and attachment of RSCs after seeding on the scaffolds for 72 h are examined by SEM. The results show that the RSCs adhered to the surface of PCL fiber and extended many pseudopodia (Figure 4). Similar results could be obtained by staining actin cytoskeleton with phalloidin (Supplementary Figure S4, Supporting Information). Furthermore, the heart, liver, spleen, lung, and kidney were harvested from the rats at week 12 postoperatively. There were no abnormalities observed from the major organs’ histological assessment (Figure 5). Taken as a whole, these results indicate that the MXene-PCL NGCs are biocompatible and could afford a beneficial microenvironment for cell growth and support nerve regeneration.

**FIGURE 6:**
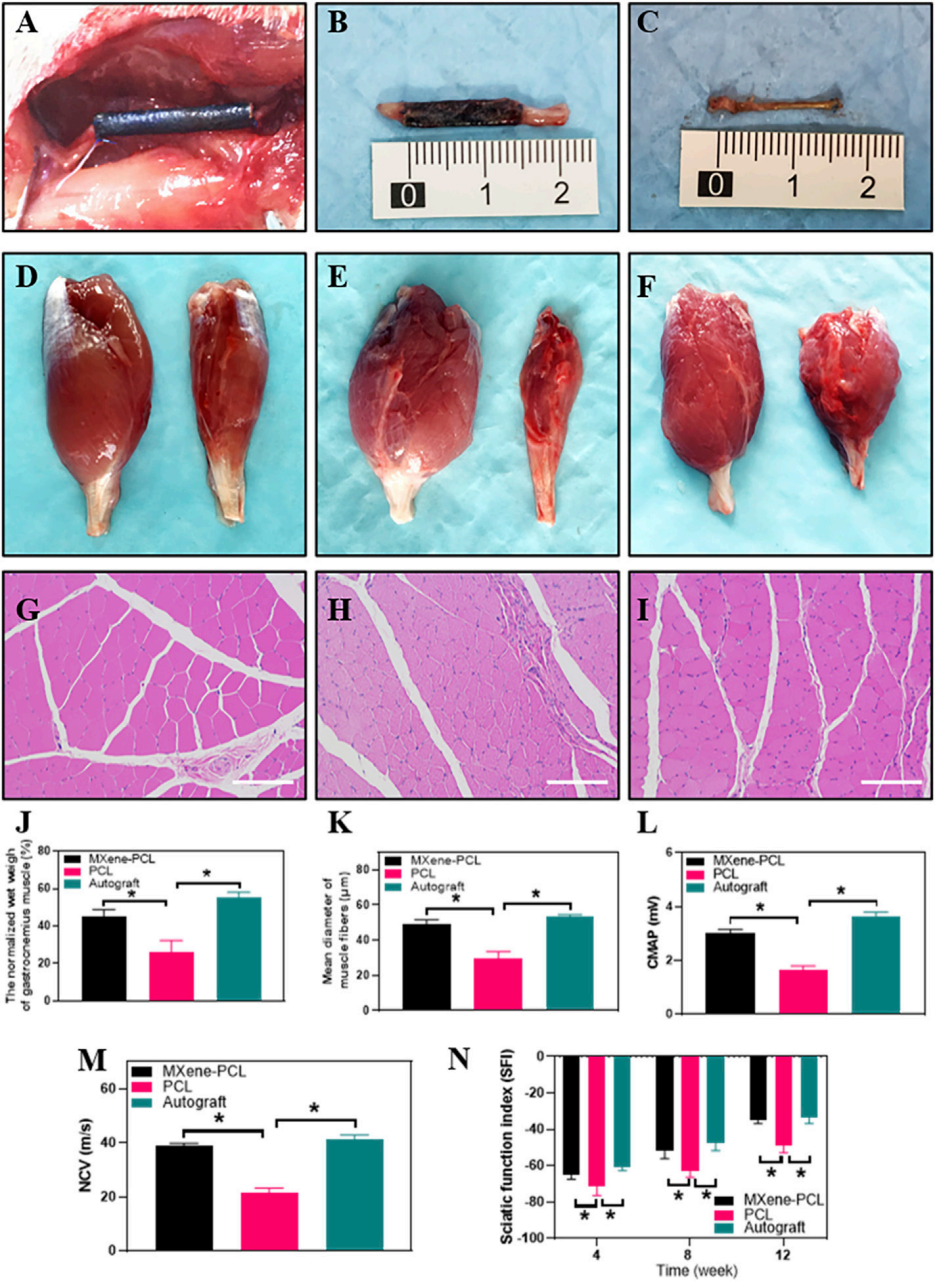
Morphology of the MXene-PCL NGC and regenerated nerve, muscle reinnervation, SFI, and electrophysiological assessment. Morphology of MXene-PCL NGC at the time of implantation **(A)**, at 12 weeks after implantation **(B)** and the regenerated nerves obtained from Mxene-PCL NGC group **(C)**. Representative optical **(D–F)** and HE staining pictures of gastrocnemius muscle at 12 weeks postoperatively **(G–I)**. The normalized wet weight of gastrocnemius muscle **(J)**, Mean diameter of muscle fibers **(K)**, compound motor action potential **(L)**, and nerve conduction velocity at 12 weeks after implantation. The sciatic functional index assessment at 4, 8 and 12 weeks after implantation. MXene-PCL group **(A–D)**, and **(G)**; PCL group **(E,H)**; Autograft group **(F,I)**. Experiments were repeated three times. *p < .05. The scale bars are 200 μm.

The modal rats were assigned into three groups randomly: PCL, autograft, and MXene-PCL groups. Postoperative assessments took place at weeks 4, 8, and 12. All rats were raised in an SPF-level environment; none was infected postoperatively. No animals showed any signs of operative complications, and the wounds healed well. At week 12, all the structures of the NGCs in animals remained intact, but the surface was degraded to a certain extent, and this can be confirmed to some extent by comparing Figure 2 and Supplementary Figure S5, Supporting Information. Although the degradation rate of PCL is very slow (Supplementary Figure S5, Supporting Information) (Lam et al., 2009), it does not affect the biocompatibility or nerve regeneration in this experiment. The pictures present the morphology of MXene-PCL NGC before and at implantation and the macroscopic view of NGCs and the regenerated nerves from different groups at 12 weeks after implantation (Figure 6, and Supplementary Figure S5, Supporting Information).

### Reinnervation of Gastrocnemius Muscle

The reinnervation of the gastrocnemius muscle was evaluated 12 weeks after implantation. The gastrocnemius muscle is an important target organ innervated by the sciatic nerve. Once the nerve damage occurs, the innervated muscles atrophy. To some extent, muscle recovery is positively correlated with nerve injury. The results show that the MXene-PCL group restored the muscle weight compared with the PCL group and showed no notable difference to that in the autograft groups. The diameters of muscle fibers in the MXene-PCL group were notably longer than that of the PCL group and showed no significant difference from that in the autograft group (Figure 6).

### Functional Recovery Evaluation of Sciatic Nerve

The SFI and electrophysiological assessment methods were conducted to evaluate the functional recovery of the sciatic nerve. The step length of each paw and the length and width of the paw prints were measured according to the footprints (Supplementary Figure S6, Supporting Information). We found that the SFI of the MXene-PCL group (−34.9) was obviously better than the PCL group (−48.8, *p* < .05) and showed no obvious difference to that of the autograft group (−33.8, *p* > .05) at postoperative 12 weeks. Electrophysiological analysis shows that the regenerated nerve CMAP and NCV of the MXene-PCL group (38.9 m s^−1^ and 3.0 mV) were notably higher than that of the PCL group (21.4 m s^−1^ and 1.6 mV, *p* < .05) and showed no notable difference from that in the autograft groups (41.28 m s^−1^ and 3.6 mV, *p* > .05) (Figure 6) at 12 weeks after implantation.

### Histological Analysis

**FIGURE 7:**
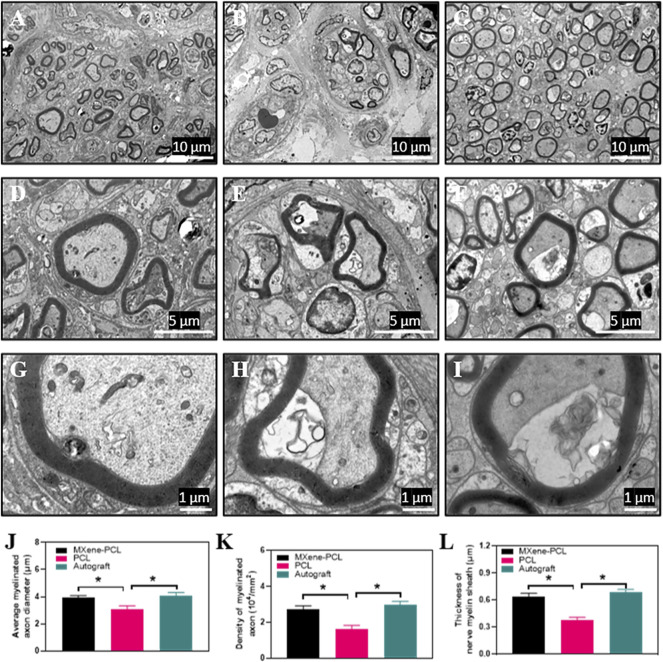
TEM images for cross-sections of regenerated nerves from the MXene-PCL conduit **(A,D,G)**, the PCL conduit **(B,E,H)**, and the autograft **(C,F,I)**, at 12 weeks postoperatively. The statistical analysis of average myelinated axon diameter **(J)**, the density of myelinated axon **(K)** and thickness of nerve myelin sheath **(L)**. All specimens were observed by TEM. Experiments were repeated three times. *p < .05. The scale bars are 10 μm **(A–C)**, 5 μm **(D–F)** and 1 μm **(G–I)**, respectively.

For validating the morphological improvement, TEM, HE, and toluidine blue staining were performed. The results reveal that the MXene-PCL contributed to the regeneration of nerve fibers and myelination compared with the PCL group. The myelin wrapped around the axon in a compact, multilayered spiral in the MXene-PCL and autograft groups (Figure 7, and Supplementary Figure S7, Supporting Information).

We found that the diameter and density of the myelinated axon, the thickness of the myelin sheaths of MXene-PCL group (3.91 μm, 2.71 × 10^4^ and 0.64 μm) showed no notable difference to that in the autograft groups (4.09 μm, 2.98 × 10^4^ and 0.685 μm), respectively (Figure 7). In contrast, only a few nerve fibers and many vacuoles appeared in the PCL group, and the diameter and density of myelinated axon myelin thickness were significantly less than that in the MXene-PCL and autograft groups.

**FIGURE 8:**
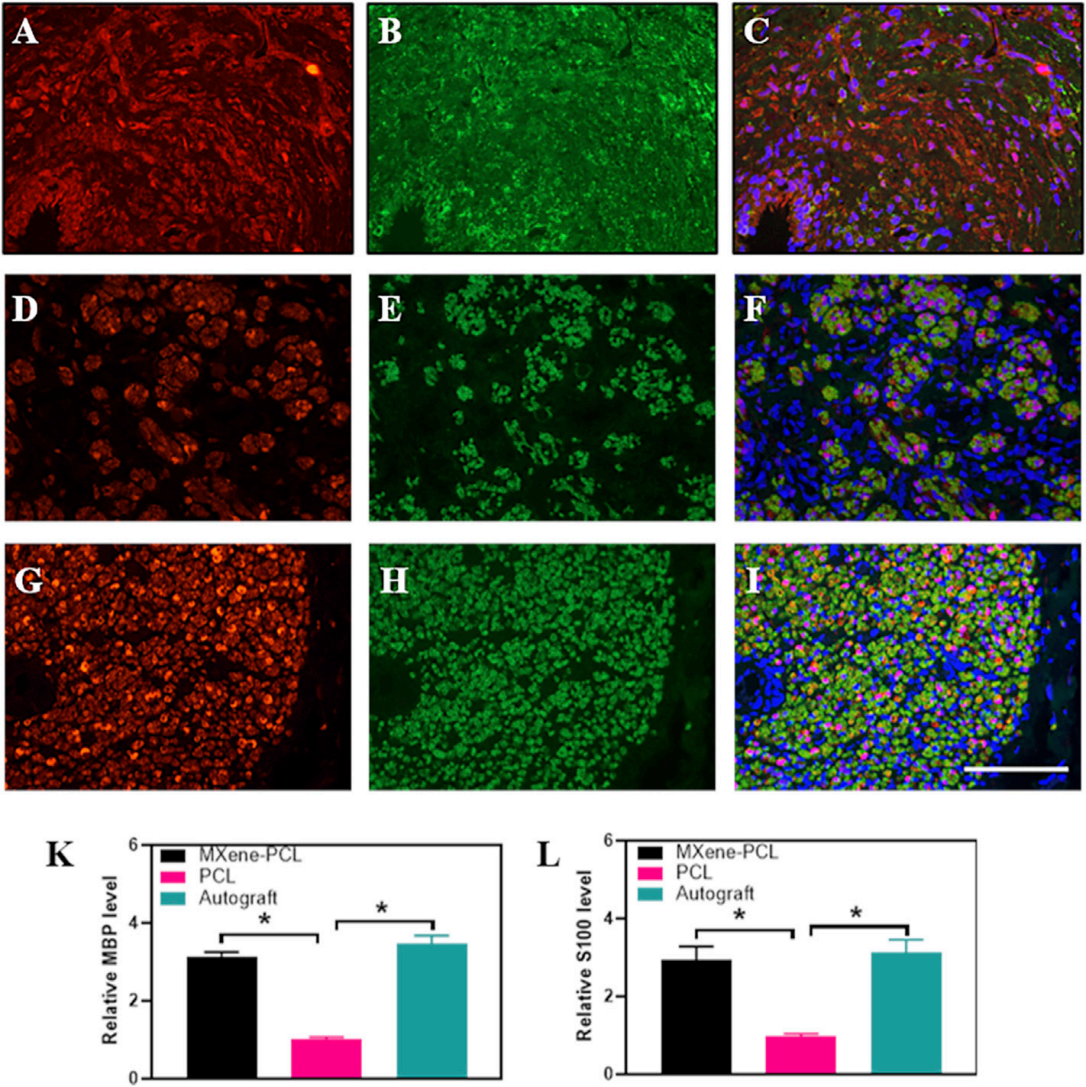
Immunofluorescence staining of S100 (red) and MBP (green) from MXene-PCL group **(A–C)**, PCL group **(D–F)**, and autograft group **(G–I)** at 12 weeks postoperatively. The Relative expression level of MBP **(K)** and S100 **(L)**. Experiments were repeated three times. *p < .05. The scale bar is 200 μm.

**FIGURE 9:**
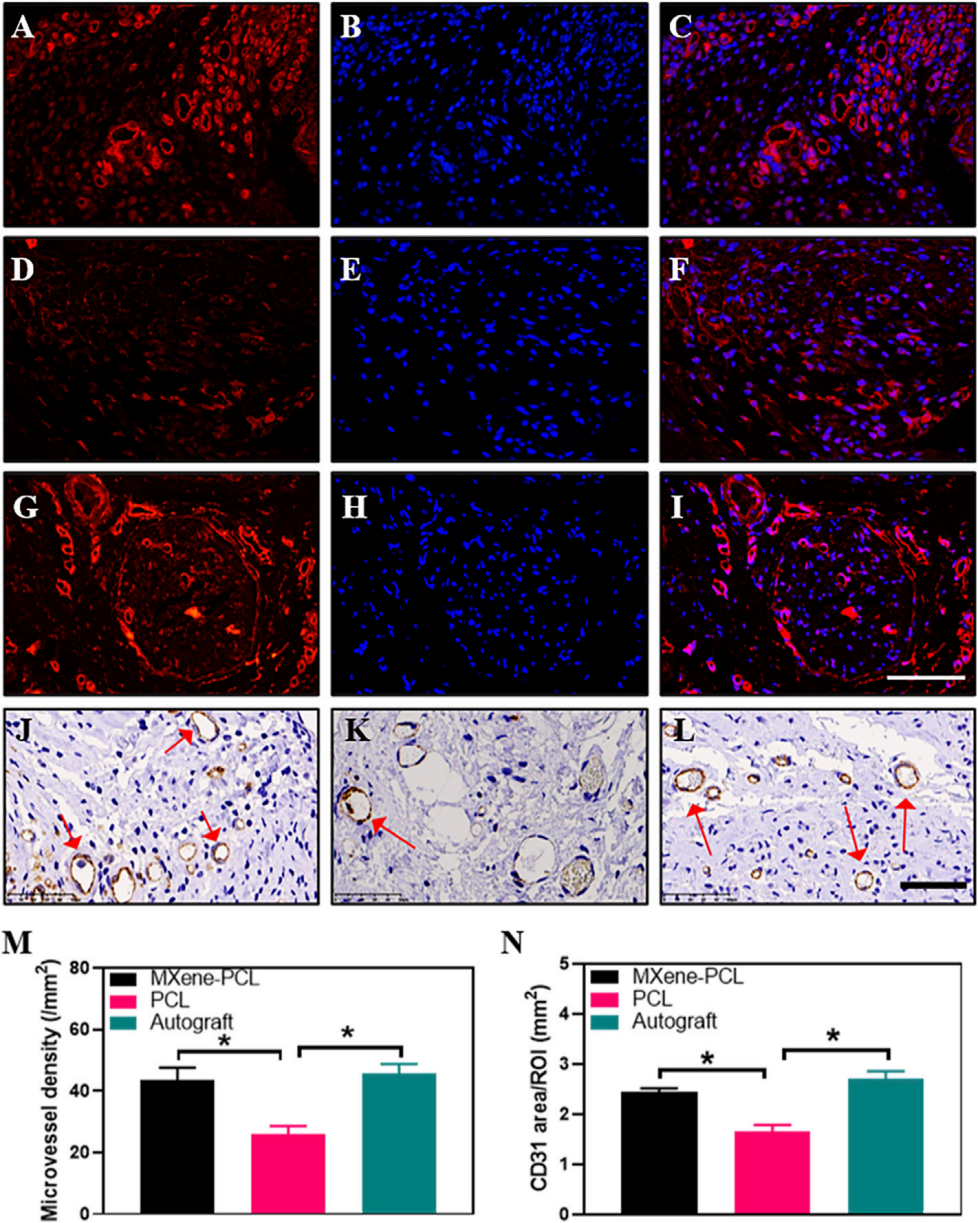
Immunofluorescence staining for CD34 (red), DAPI (blue), and Immunohistochemistry staining for CD31 from MXene-PCL group **(A–C,J)**, PCL group **(D–F,K)**, and autograft group **(G–I,L)** at 12 weeks postoperatively. Microvessel density was calculated from CD34 staining **(M)**. CD31 area was calculated from CD31 staining **(N)**. Experiments were repeated three times. *p < .05. The scale bars are 100 μm **(A–I)**, and 50 μm **(J–L)**.

To evaluate the effect of the electroconductive MXene-PCL NGC on nerve regeneration, the Schwann cell marker protein 100 (S100) and neuron-specific protein (MBP) and the typical axon protein of neurofilament protein 200 (NF200) and β-III-tubulin (Tuj 1) were evaluated by immunofluorescence staining. The results reveal that the expression level of MBP and S100 in the MXene-PCL group was 3.12 and 2.93 times higher than that in the PCL group, respectively (*p* < .05) while 3.48 and 2.93 times higher in the autograft group than that in the PCL group. The expression level of NF200 in the MXene-PCL and autograft groups was 2.16 and 2.90 times higher than that in PCL group. The expression level of Tuj 1 in the MXene-PCL and autograft groups was 3.40 and 4.22 times of the PCL group (*p* < .05). The above results confirmed that the MXene-PCL NGC effectively improved the myelination and regeneration of the nerve fibers with a similar effect to the autograft nerve (Figure 8, and Supplementary Figure S8, Supporting Information). Taking a step further, we evaluated the neovascularization by immunohistochemistry of specific protein markers of endothelial cells (CD31) and immunofluorescence of hematopoietic progenitor cell markers (CD34). It was found that the MXene-PCL and autograft groups showed more vascular-like structures than that of the PCL group by nerve cross-section staining. The average microvessel density calculated from CD34 and CD31 areas of the MXene-PCL group (43.67 mm^−2^ and 2.44 mm^2^) were notably higher than that of the PCL group (26.00 mm^−2^ and 1.64 mm^2^, *p* < .05) and showed no notable difference from that in the autograft groups (45.67 mm^−2^ and 2.71 mm^2^, *p* > .05) at 12 weeks postoperative (Figure 9).

## Discussion

Promoting nerve regeneration and transmitting electrophysiological signals are two important aspects for the repair of peripheral nerve defects (Zhao et al., 2020; Kasper et al., 2021; Kunisaki et al., 2021). The fabricated MXene-PCL NGC by electrospinning could provide suitable mechanical support, affording a necessary lumen for nerve regeneration. In addition, the nerve signal is a kind of electrical signal, the transmission of the electrical signal from upstream to downstream, and forming complete feedback is indispensable to exert its function (Silverå Ejneby et al., 2021). Ti_3_C_2_T_x_ MXene is hailed as a new two-dimensional material to rival graphene. It has gradually become a research hot spot in the field of electronic biosensing and tissue engineering due to its excellent characteristics, such as decent biocompatibility, large specific surface area, good electrical conductivity, and hydrophilicity (Mao et al., 2020; Rui Li et al., 2021; Zhou et al., 2021). The facile fabrication process of the MXene-PCL NGC manufacturing process (just using the spraying method) made it easy to transformed for further applications. There are many reports on its application of probing neural activity and artificial synapse (Xu et al., 2016; Wang et al., 2021; Wei et al., 2021). Ti_3_C_2_T_x_ MXene could not only promote proliferation and differentiation in BMSCs, but also transmit electric signals and accelerate tissue regeneration (Basara et al., 2020; Huang et al., 2020; Mao et al., 2020; Rastin et al., 2020). It has never been applied as an electroactive polymer for nerve regeneration *in vivo*. Here, we engineered a MXene-PCL NGC by using electrospinning and spray-coated technology; the prepared MXene-PCL membrane showed high conductivity (0.03 S/cm). Prior to *in vivo* experiments, the cell viability and affinity of the prepared materials were evaluated using RSCs cells. As anticipated, the obtained MXene-PCL conduct could afford a beneficial environment for RSC adhesion and proliferation with good cell affinity.

In addition, a 15-mm long sciatic nerve defect SD rat model was used to investigate its repair effect on nerve regeneration *in vivo*. The nerve defect was sutured with NGC or autologous nerve. The MXene-PCL NGC showed similar results with the autograft in sciatic function index, electrophysiological examination, and morphological nerve regeneration at 12 weeks postoperative. The conductive MXene-PCL NGC could transmit physiological neural electrical signals, facilitate angiogenesis, and feed to stimulate nerve regeneration. Taken together, the MXene-PCL NGC revealed better effects in promoting nerve regeneration due to the cooperation of multiple other factors: a suitable mechanical support, affording a necessary lumen (Gryshkov et al., 2021; Qian et al., 2021b), conducting of nerve signals from upstream to downstream, and forming complete feedback. As shown in Figure 1, the MXene-NGC could restore physiological nerve signal transduction from upstream to downstream and form complete biofeedback to the proximal and distal nerve stumps. The bioelectrical properties of cell membranes could create a vulnerable local electric field, the free electrons from the environment could create certain current through conductive material, and in our study, the additional application of conductive MXene would enhance the electric field, which, in turn, could promote cell proliferation under the interactive stimuli from surrounding cells (Heo et al., 2011; Park et al., 2011; Guo et al., 2016; Jin et al., 2021).

The conductive MXene-PCL NGC matched well with the electrophysiological properties of the sciatic nerve; meanwhile, a higher signal of CD34 and CD31 indicated the electrical signals could stimulate neovascularization. In turn, the vascular would strengthen the nutritional supply for neural functional reconstruction (Saio et al., 2021; Thibodeau et al., 2021; Wu et al., 2021; Xiaobin Li et al., 2021). The conductive MXene NGC showed a better effect in nerve regeneration than nonconductive NGC. Said another way, the conductive MXene-PCL NGC could transmit physiological neural electrical signals, creating a good microenvironment for nerve regeneration, and feeding to stimulate nerve regeneration (Zhou et al., 2016; Jin et al., 2021).

This paper presents a novel design of a MXene-PCL NGC that could transmit self-originated electrical stimulation. In the future, it can be combined with other features to promote nerve regeneration.

## Conclusion

In summary, we prepared a conductive MXene-PCL NGC for the repair of PNI. Our data confirms that NGC supports well the attachment and proliferation of RSCs. In addition, the NGC could transmit physiological neural electrical signals and increase angiogenesis. The MXene-PCL NGC could transmit physiologic nervous electric signals, creating a good microenvironment for nerve regeneration and feeding to stimulate nerve regeneration. The design that the conductive layer was coated outside the conduit rather than inside could avoid the potential toxicity caused by direct contact between the MXene particles and newly regenerated nerves, and it is easy to prepare the MXene-PCL NGC just using the spraying method. In the near-term future, we desired to further reveal the network between electrical signals and nerve regeneration.

## Data Availability

The original contributions presented in the study are included in the article/Supplementary Material, further inquiries can be directed to the corresponding authors.

## References

[B1] Andón F. T. Fadeel B. (2013). Programmed Cell Death: Molecular Mechanisms and Implications for Safety Assessment of Nanomaterials. Acc. Chem. Res. 46, 733–742. 10.1021/ar300020b 22720979

[B2] Barroca N. Marote A. Vieira S. I. Almeida A. Fernandes M. H. V. Vilarinho P. M. (2018). Electrically Polarized PLLA Nanofibers as Neural Tissue Engineering Scaffolds with Improved Neuritogenesis. Colloids Surf. B: Biointerfaces 167, 93–103. 10.1016/j.colsurfb.2018.03.050 29627682

[B3] Basara G. Saeidi-Javash M. Ren X. Bahcecioglu G. Wyatt B. C. Anasori B. (2022). Electrically Conductive 3D Printed Ti3C2T MXene-PEG Composite Constructs for Cardiac Tissue Engineering. Acta Biomater. 139, 179–189. 10.1016/j.actbio.2020.12.033 33352299 PMC8213874

[B4] Black M. M. Lasek R. J. (1979). Slowing of the Rate of Axonal Regeneration during Growth and Maturation. Exp. Neurol. 63, 108–119. 10.1016/0014-4886(79)90188-2 467539

[B5] Cao W.-T. Feng W. Jiang Y.-Y. Ma C. Zhou Z.-F. Ma M.-G. (2019a). Two-dimensional MXene-Reinforced Robust Surface Superhydrophobicity with Self-Cleaning and Photothermal-Actuating Binary Effects. Mater. Horiz. 6, 1057–1065. 10.1039/C8MH01566J

[B6] Cao W. T. Ma C. Mao D. S. Zhang J. Ma M. G. Chen F. (2019b). MXene-Reinforced Cellulose Nanofibril Inks for 3D-Printed Smart Fibres and Textiles. Adv. Funct. Mater. 29, 1905898. 10.1002/adfm.201905898

[B7] Dervan A. Franchi A. Almeida-Gonzalez F. R. Dowling J. K. Kwakyi O. B. Mccoy C. E. (2021). Biomaterial and Therapeutic Approaches for the Manipulation of Macrophage Phenotype in Peripheral and Central Nerve Repair. Pharmaceutics 13, 2161. 10.3390/pharmaceutics13122161 34959446 PMC8706646

[B8] Foster C. H. Karsy M. Jensen M. R. Guan J. Eli I. Mahan M. A. (2019). Trends and Cost-Analysis of Lower Extremity Nerve Injury Using the National Inpatient Sample. Neurosurg. 85, 250–256. 10.1093/neuros/nyy265 29889258

[B9] Fregnan F. Muratori L. Bassani G. A. Crosio A. Biagiotti M. Vincoli V. (2020). Preclinical Validation of SilkBridgeTM for Peripheral Nerve Regeneration. Front. Bioeng. Biotechnol. 8, 835. 10.3389/fbioe.2020.00835 32850714 PMC7426473

[B10] Gryshkov O. Al Halabi F. Kuhn A. I. Leal-Marin S. Freund L. J. Förthmann M. (2021). PVDF and P(VDF-TrFE) Electrospun Scaffolds for Nerve Graft Engineering: A Comparative Study on Piezoelectric and Structural Properties, and *In Vitro* Biocompatibility. Int. J. Mol. Sci. 22, 11373. 10.3390/ijms222111373 34768804 PMC8583857

[B11] Guo R. Zhang S. Xiao M. Qian F. He Z. Li D. (2016). Accelerating Bioelectric Functional Development of Neural Stem Cells by Graphene Coupling: Implications for Neural Interfacing with Conductive Materials. Biomaterials 106, 193–204. 10.1016/j.biomaterials.2016.08.019 27566868

[B12] Hao P. Yang Z.-Y. Li X.-G. Liu F.-D. Duan H.-M. Hao F. (2022). Biomimetic Chitosan Scaffolds with Long-Term Controlled Release of Nerve Growth Factor Repairs 20-Mm-Long Sciatic Nerve Defects in Rats. Neural Regen. Res. 17, 1146–1155. 10.4103/1673-5374.324860 34558544 PMC8552858

[B13] Heo C. Yoo J. Lee S. Jo A. Jung S. Yoo H. (2011). The Control of Neural Cell-To-Cell Interactions through Non-contact Electrical Field Stimulation Using Graphene Electrodes. Biomaterials 32, 19–27. 10.1016/j.biomaterials.2010.08.095 20880583

[B14] Huang R. Chen X. Dong Y. Zhang X. Wei Y. Yang Z. (2020). MXene Composite Nanofibers for Cell Culture and Tissue Engineering. ACS Appl. Bio Mater. 3, 2125–2131. 10.1021/acsabm.0c00007 35025264

[B15] Jin F. Li T. Yuan T. Du L. Lai C. Wu Q. (2021). Physiologically Self-Regulated, Fully Implantable, Battery-free System for Peripheral Nerve Restoration. Adv. Mater. 33, 2104175. 10.1002/adma.202104175 34608668

[B16] Karsy M. Watkins R. Jensen M. R. Guan J. Brock A. A. Mahan M. A. (2019). Trends and Cost Analysis of Upper Extremity Nerve Injury Using the National (Nationwide) Inpatient Sample. World Neurosurg. 123, e488–e500. 10.1016/j.wneu.2018.11.192 30502477

[B17] Kasper M. Ellenbogen B. Hardy R. Cydis M. Mojica-Santiago J. Afridi A. (2021). Development of a Magnetically Aligned Regenerative Tissue-Engineered Electronic Nerve Interface for Peripheral Nerve Applications. Biomaterials 279, 121212. 10.1016/j.biomaterials.2021.121212 34717196 PMC9036633

[B18] Kunisaki A. Kodama A. Ishikawa M. Ueda T. Lima M. D. Kondo T. (2021). Carbon-nanotube Yarns Induce Axonal Regeneration in Peripheral Nerve Defect. Sci. Rep. 11, 19562. 10.1038/s41598-021-98603-7 34599218 PMC8486759

[B19] Lam C. X. F. Hutmacher D. W. Schantz J.-T. Woodruff M. A. Teoh S. H. (2009). Evaluation of Polycaprolactone Scaffold Degradation for 6 Monthsin Vitroandin Vivo. J. Biomed. Mater. Res. 90A, 906–919. 10.1002/jbm.a.32052 18646204

[B20] Mao L. Hu S. Gao Y. Wang L. Zhao W. Fu L. (2020). Biodegradable and Electroactive Regenerated Bacterial Cellulose/MXene (Ti 3 C 2 T X ) Composite Hydrogel as Wound Dressing for Accelerating Skin Wound Healing under Electrical Stimulation. Adv. Healthc. Mater. 9, 2000872. 10.1002/adhm.202000872 32864898

[B21] Park S. Y. Park J. Sim S. H. Sung M. G. Kim K. S. Hong B. H. (2011). Enhanced Differentiation of Human Neural Stem Cells into Neurons on Graphene. Adv. Mater. 23, H263–H267. 10.1002/adma.201101503 21823178

[B22] Qian Y. Cheng Y. Cai J. Zhao X. Ouyang Y. Yuan W.-E. (2019). Advances in Electrical and Magnetic Stimulation on Nerve Regeneration. Regenerative Med. 14, 969–979. 10.2217/rme-2018-0079 31583954

[B23] Qian Y. Cheng Y. Song J. Xu Y. Yuan W. E. Fan C. (2020). Mechano-Informed Biomimetic Polymer Scaffolds by Incorporating Self-Powered Zinc Oxide Nanogenerators Enhance Motor Recovery and Neural Function. Small 16, 2000796. 10.1002/smll.202000796 32633072

[B24] Qian Y. Song J. Zhao X. Chen W. Ouyang Y. Yuan W. (2018a). 3D Fabrication with Integration Molding of a Graphene Oxide/Polycaprolactone Nanoscaffold for Neurite Regeneration and Angiogenesis. Adv. Sci. 5, 1700499. 10.1002/advs.201700499 PMC5908351 29721407

[B25] Qian Y. Zhao X. Han Q. Chen W. Li H. Yuan W. (2018b). An Integrated Multi-Layer 3D-Fabrication of PDA/RGD Coated Graphene Loaded PCL Nanoscaffold for Peripheral Nerve Restoration. Nat. Commun. 9, 323. 10.1038/s41467-017-02598-7 29358641 PMC5778129

[B26] Qian Y. Lin H. Yan Z. Shi J. Fan C. (2021a). Functional Nanomaterials in Peripheral Nerve Regeneration: Scaffold Design, Chemical Principles and Microenvironmental Remodeling. Mater. Today 51, 165–187. 10.1016/j.mattod.2021.09.014

[B27] Qian Y. Wang X. Song J. Chen W. Chen S. Jin Y. (2021b). Preclinical Assessment on Neuronal Regeneration in the Injury-Related Microenvironment of Graphene-Based Scaffolds. NPJ Regen. Med. 6, 31. 10.1038/s41536-021-00142-2 34078912 PMC8172906

[B28] Rastin H. Zhang B. Mazinani A. Hassan K. Bi J. Tung T. T. (2020). 3D Bioprinting of Cell-Laden Electroconductive MXene Nanocomposite Bioinks. Nanoscale 12, 16069–16080. 10.1039/d0nr02581j 32579663

[B29] Rui Li R. Xu J. Rao Z. Deng R. Xu Y. Qiu S. (2021). Facilitate Angiogenesis and Neurogenesis by Growth Factors Integrated Decellularized Matrix Hydrogel. Tissue Eng. A 27, 771–787. 10.1089/ten.TEA.2020.0227 33107410

[B30] Saio S. Konishi K. Hohjoh H. Tamura Y. Masutani T. Iddamalgoda A. (2021). Extracellular Environment-Controlled Angiogenesis, and Potential Application for Peripheral Nerve Regeneration. Int. J. Mol. Sci. 22, 11169. 10.3390/ijms222011169 34681829 PMC8541280

[B31] Senger J.-L. B. Rabey K. N. Acton L. Lin Y.-H. S. Lingrell S. Chan K. M. (2021). Recovering the Regenerative Potential in Chronically Injured Nerves by Using Conditioning Electrical Stimulation. J. Neurosurg., 1–13. 10.3171/2021.4.jns21398 34653977

[B32] Shen J. Wang J. Liu X. Sun Y. Yin A. Chai Y. (2021). *In Situ* Prevascularization Strategy with Three-Dimensional Porous Conduits for Neural Tissue Engineering. ACS Appl. Mater. Inter. 13, 50785–50801. 10.1021/acsami.1c16138 34664947

[B33] Shin S. R. Li Y.-C. Jang H. L. Khoshakhlagh P. Akbari M. Nasajpour A. (2016). Graphene-based Materials for Tissue Engineering. Adv. Drug Deliv. Rev. 105, 255–274. 10.1016/j.addr.2016.03.007 27037064 PMC5039063

[B34] Silverå Ejneby M. Jakešová M. Ferrero J. J. Migliaccio L. Sahalianov I. Zhao Z. (2021). Chronic Electrical Stimulation of Peripheral Nerves via Deep-Red Light Transduced by an Implanted Organic Photocapacitor. Nat. Biomed. Eng. 10.1038/s41551-021-00817-7 34916610

[B35] Song S. Mcconnell K. W. Amores D. Levinson A. Vogel H. Quarta M. (2021). Electrical Stimulation of Human Neural Stem Cells via Conductive Polymer Nerve Guides Enhances Peripheral Nerve Recovery. Biomaterials 275, 120982. 10.1016/j.biomaterials.2021.120982 34214785 PMC8325644

[B36] Sun X. Bai Y. Zhai H. Liu S. Zhang C. Xu Y. (2019). Devising Micro/nano-Architectures in Multi-Channel Nerve Conduits towards a Pro-regenerative Matrix for the Repair of Spinal Cord Injury. Acta Biomater. 86, 194–206. 10.1016/j.actbio.2018.12.032 30586646

[B37] Sun Y. Chi X. Meng H. Ma M. Wang J. Feng Z. (2021). Polylysine-decorated Macroporous Microcarriers Laden with Adipose-Derived Stem Cells Promote Nerve Regeneration *In Vivo* . Bioactive Mater. 6, 3987–3998. 10.1016/j.bioactmat.2021.03.029 PMC8082165 33997488

[B38] Thibodeau A. Galbraith T. Fauvel C. M. Khuong H. T. Berthod F. (2022). Repair of Peripheral Nerve Injuries Using a Prevascularized Cell-Based Tissue-Engineered Nerve Conduit. Biomaterials 280, 121269. 10.1016/j.biomaterials.2021.121269 34847434

[B39] Wang Y. Wang X. Li X. Bai Y. Xiao H. Liu Y. (2019). Engineering 3D Ion Transport Channels for Flexible MXene Films with superior Capacitive Performance. Adv. Funct. Mater. 29, 1900326. 10.1002/adfm.201900326

[B40] Wang Y. Zhang Y. Zhang Z. Su Y. Wang Z. Dong M. (2020). An Injectable High-Conductive Bimaterial Scaffold for Neural Stimulation. Colloids Surf. B: Biointerfaces 195, 111210. 10.1016/j.colsurfb.2020.111210 32679447

[B41] Wang K. Jia Y. Yan X. (2021). Neuro-Receptor Mediated Synapse Device Based on Crumpled MXene Ti 3 C 2 T X Nanosheets. Adv. Funct. Mater. 31, 2104304. 10.1002/adfm.202104304

[B42] Wang B. Kou Y.-H. Jiang B.-G. Lu C.-F. Liu Z.-Y. Han S. (2022). Chitin Scaffold Combined with Autologous Small Nerve Repairs Sciatic Nerve Defects. Neural Regen. Res. 17, 1106–1114. 10.4103/1673-5374.324859 34558539 PMC8552871

[B43] Wei H. Yu H. Gong J. Ma M. Han H. Ni Y. (2021). Redox MXene Artificial Synapse with Bidirectional Plasticity and Hypersensitive Responsibility. Adv. Funct. Mater. 31, 2007232. 10.1002/adfm.202007232

[B44] Wu P. Tong Z. Luo L. Zhao Y. Chen F. Li Y. (2021). Comprehensive Strategy of Conduit Guidance Combined with VEGF Producing Schwann Cells Accelerates Peripheral Nerve Repair. Bioactive Mater. 6, 3515–3527. 10.1016/j.bioactmat.2021.03.020 PMC8008177 33842738

[B45] Xiaobin Li X. He L. Li Y. Chao M. Li M. Wan P. (2021). Healable, Degradable, and Conductive MXene Nanocomposite Hydrogel for Multifunctional Epidermal Sensors. ACS Nano 15, 7765–7773. 10.1021/acsnano.1c01751 33769046

[B46] Xu B. Zhu M. Zhang W. Zhen X. Pei Z. Xue Q. (2016). Ultrathin MXene-Micropattern-Based Field-Effect Transistor for Probing Neural Activity. Adv. Mater. 28, 3333–3339. 10.1002/adma.201504657 26924616

[B47] Yan J. Wu R. Liao S. Jiang M. Qian Y. (2020). Applications of Polydopamine-Modified Scaffolds in the Peripheral Nerve Tissue Engineering. Front. Bioeng. Biotechnol. 8, 590998. 10.3389/fbioe.2020.590998 33195158 PMC7609920

[B48] Yoo J. Park J. H. Kwon Y. W. Chung J. J. Choi I. C. Nam J. J. (2020). Augmented Peripheral Nerve Regeneration through Elastic Nerve Guidance Conduits Prepared Using a Porous PLCL Membrane with a 3D Printed Collagen Hydrogel. Biomater. Sci. 8, 6261–6271. 10.1039/d0bm00847h 33016275

[B49] Yu J. Lin Y. Wang G. Song J. Hayat U. Liu C. (2022). Zein-induced Immune Response and Modulation by Size, Pore Structure and Drug-Loading: Application for Sciatic Nerve Regeneration. Acta Biomater. 140, 289–301. 10.1016/j.actbio.2021.11.035 34843952

[B50] Zeng W. Hui H. Liu Z. Chang Z. Wang M. He B. (2021). TPP Ionically Cross-Linked Chitosan/PLGA Microspheres for the Delivery of NGF for Peripheral Nerve System Repair. Carbohydr. Polym. 258, 117684. 10.1016/j.carbpol.2021.117684 33593557

[B51] Zhang D. Yao Y. Duan Y. Yu X. Shi H. Nakkala J. R. (2020). Surface-Anchored Graphene Oxide Nanosheets on Cell-Scale Micropatterned Poly(d,l-Lactide-Co-Caprolactone) Conduits Promote Peripheral Nerve Regeneration. ACS Appl. Mater. Inter. 12, 7915–7930. 10.1021/acsami.9b20321 31935055

[B52] Zhang J. Zhang X. Wang C. Li F. Qiao Z. Zeng L. (2021). Conductive Composite Fiber with Optimized Alignment Guides Neural Regeneration under Electrical Stimulation. Adv. Healthc. Mater. 10, 2000604. 10.1002/adhm.202000604 33300246

[B53] Zhang Y. Li X. Liang J. Luo Y. Tang N. Ye S. (2022). Microcystis Aeruginosa's Exposure to an Antagonism of Nanoplastics and MWCNTs: The Disorders in Cellular and Metabolic Processes. Chemosphere 288, 132516. 10.1016/j.chemosphere.2021.132516 34648785

[B54] Zhao Y. Liang Y. Ding S. Zhang K. Mao H.-q. Yang Y. (2020). Application of Conductive PPy/SF Composite Scaffold and Electrical Stimulation for Neural Tissue Engineering. Biomaterials 255, 120164. 10.1016/j.biomaterials.2020.120164 32554132

[B55] Zhou Z.-F. Zhang F. Wang J.-G. Chen Q.-C. Yang W.-Z. He N. (2016). Electrospinning of PELA/PPY Fibrous Conduits: Promoting Peripheral Nerve Regeneration in Rats by Self-Originated Electrical Stimulation. ACS Biomater. Sci. Eng. 2, 1572–1581. 10.1021/acsbiomaterials.6b00335 33440592

[B56] Zhou L. Zheng H. Liu Z. Wang S. Liu Z. Chen F. (2021). Conductive Antibacterial Hemostatic Multifunctional Scaffolds Based on Ti3C2Tx MXene Nanosheets for Promoting Multidrug-Resistant Bacteria-Infected Wound Healing. ACS Nano 15, 2468–2480. 10.1021/acsnano.0c06287 33565857

