# Supplementary material for: Ti3C2Tx MXene-Coated Electrospun PCL Conduits for Enhancing Neurite Regeneration and Angiogenesis
Source: Front Bioeng Biotechnol. 2022 Mar 16;10:850650. doi: 10.3389/fbioe.2022.850650 (PMC8966647; doi:10.3389/fbioe.2022.850650)
Supplement: Supplementary file 1 [file DataSheet1.docx]

Supporting Information


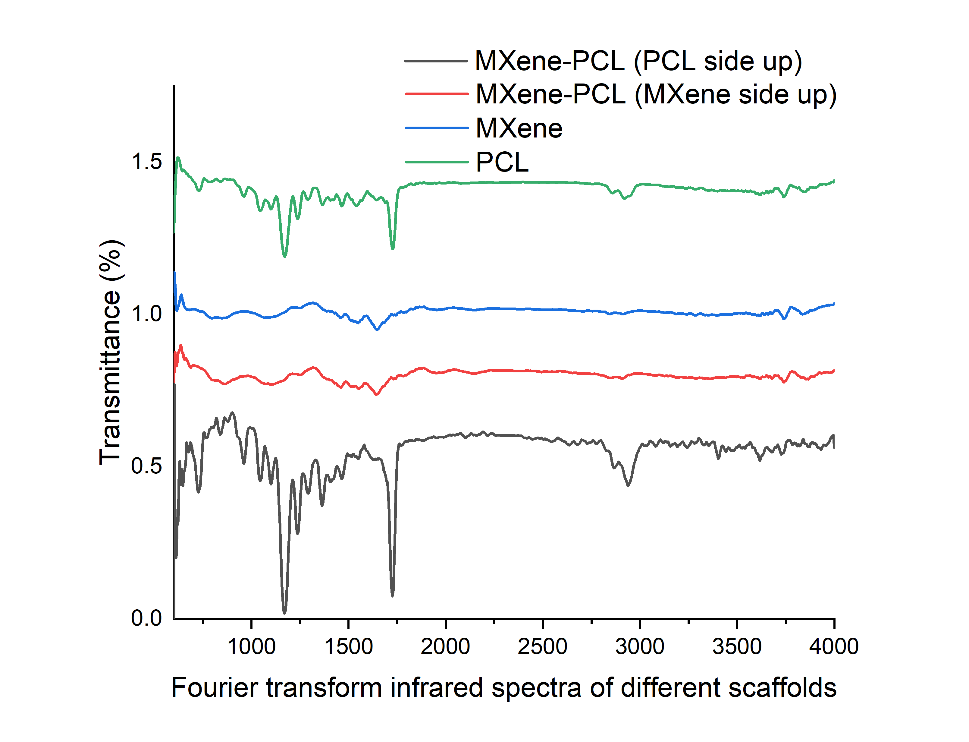


**Fig. S1.** Fourier transform infrared spectra of materials prepared and used in the study.


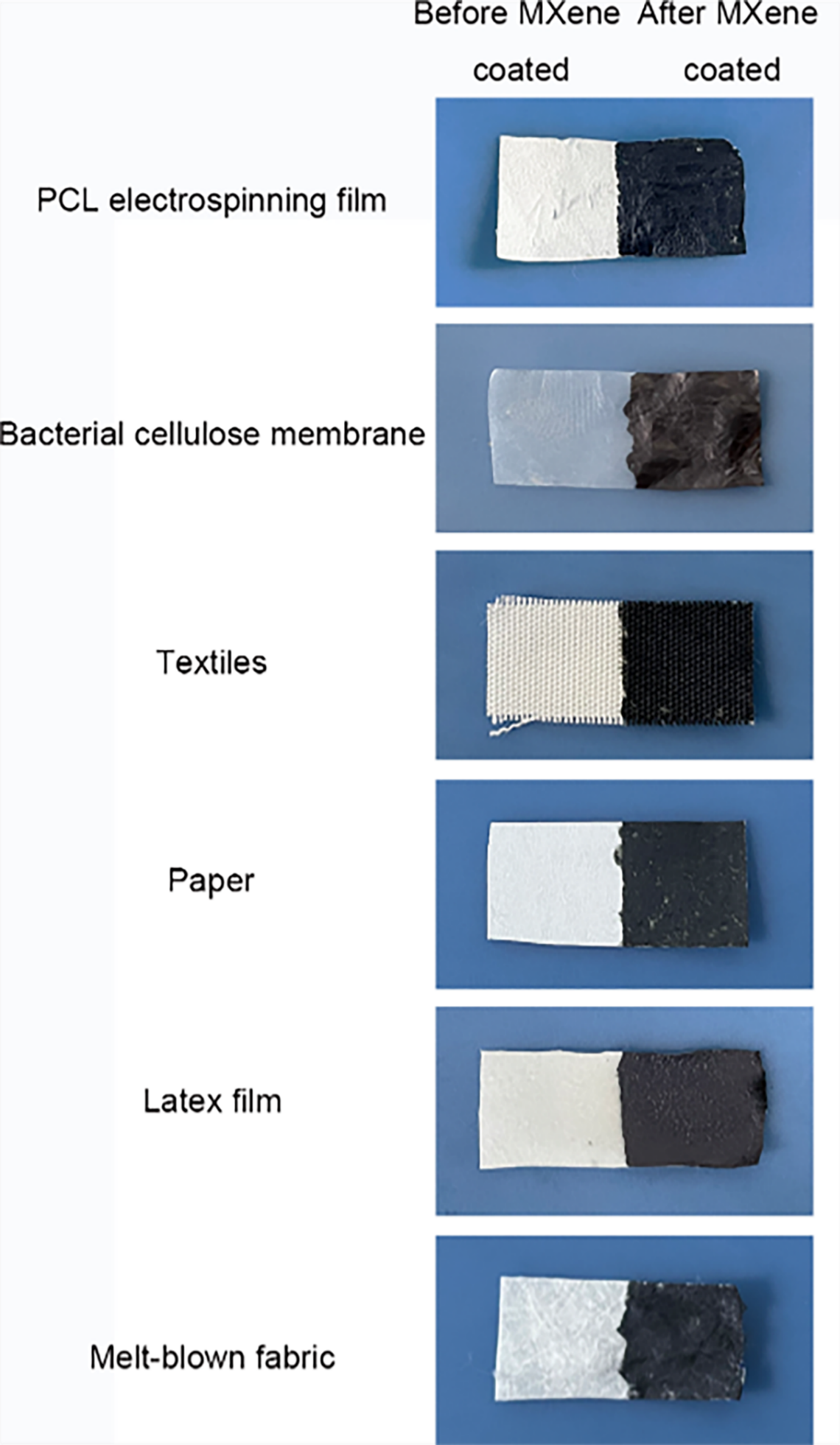


**Fig. S2.** The photographs of MXene coated on a variety of materials.


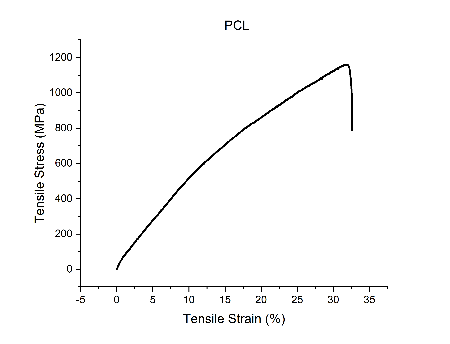


**B**


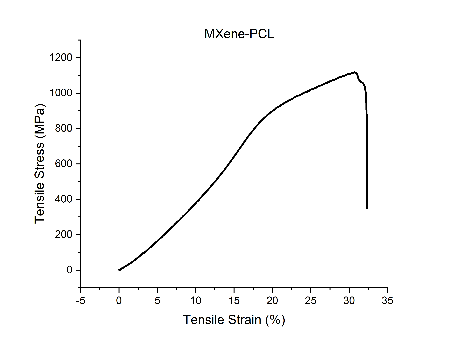


**A**

**Fig. S3.** Stress-strain curves of Mxene-PCL A) and PCL B) NGC fibrous meshes.


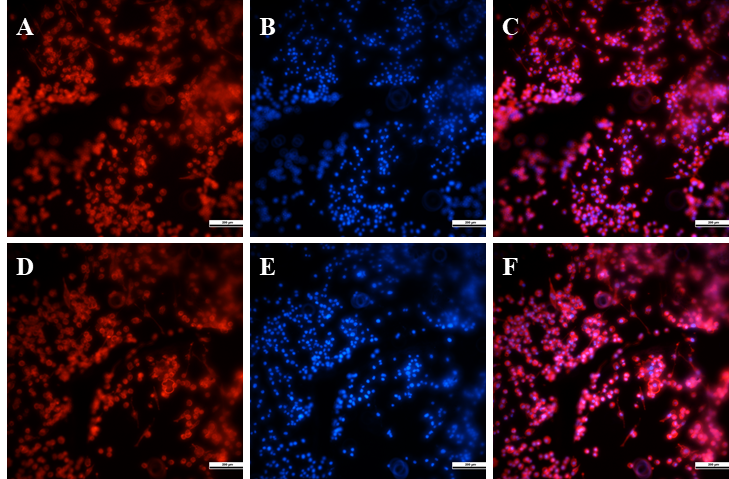


**Fig. S4.** Observation of RSC morphology on PCL side of Mxene-PCL and PCL NGC scaffolds through immunofluorescence staining for phalloidin. RSCs were examined by immunofluorescence staining for phalloidin after seeded on the scaffolds for 72 h. Mxene-PCL scaffold A–C) and PCL scaffold D–F). The scale bar is 200 μm.


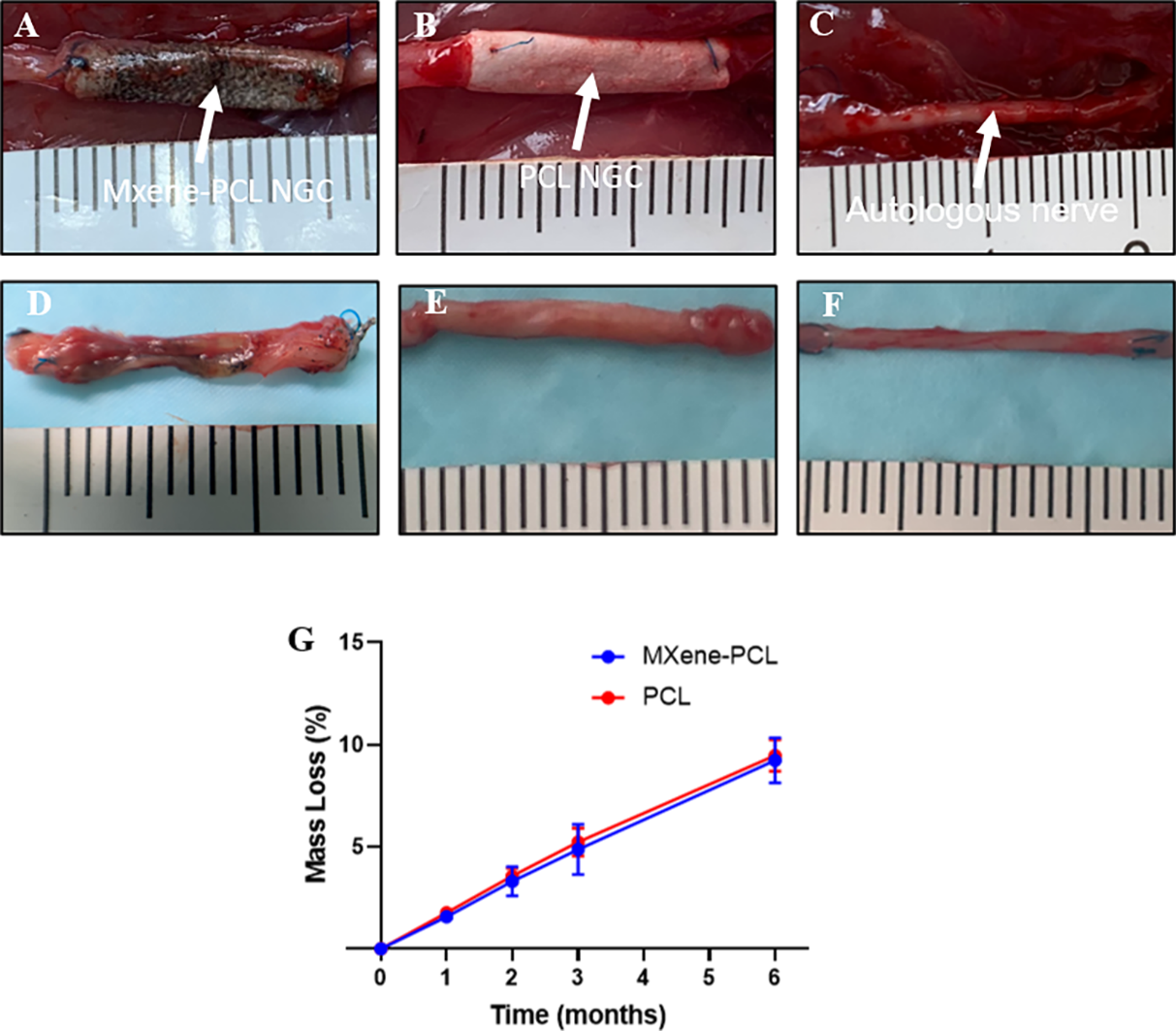


**Fig. S5.** Macroscopic view of different NGCs and regenerative nerve at 12 weeks after implantation. The nerve defect was sutured with Mxene-PCL conduit A), PCL conduit B), and autologous nerve graft C). The newborn rat nerves obtained from the Mxene-PCL group D), the PCL group E), the autograft group F). and the in vitro degradation performance of the conduit G).


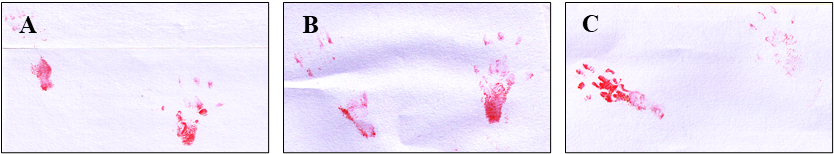


**Fig. S6.** Footprints from MXene-PCL group A), PCL group B), and autograft group C) at 12 weeks postoperatively.


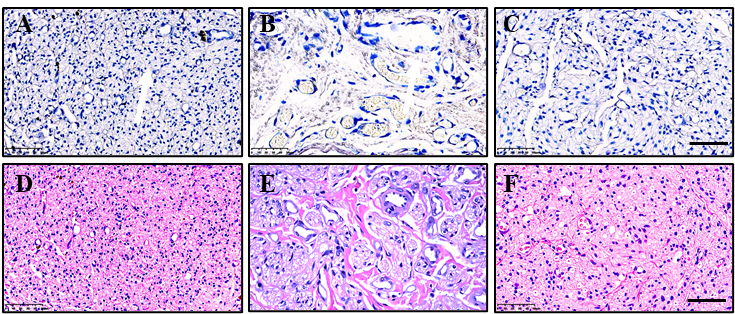


**Fig. S7.** Toluidine blue and HE staining of regenerated nerve fibers from MXene-PCL group A), PCL group B), and autograft group C) at 12 weeks postoperatively. The scale bars are 50 μm.


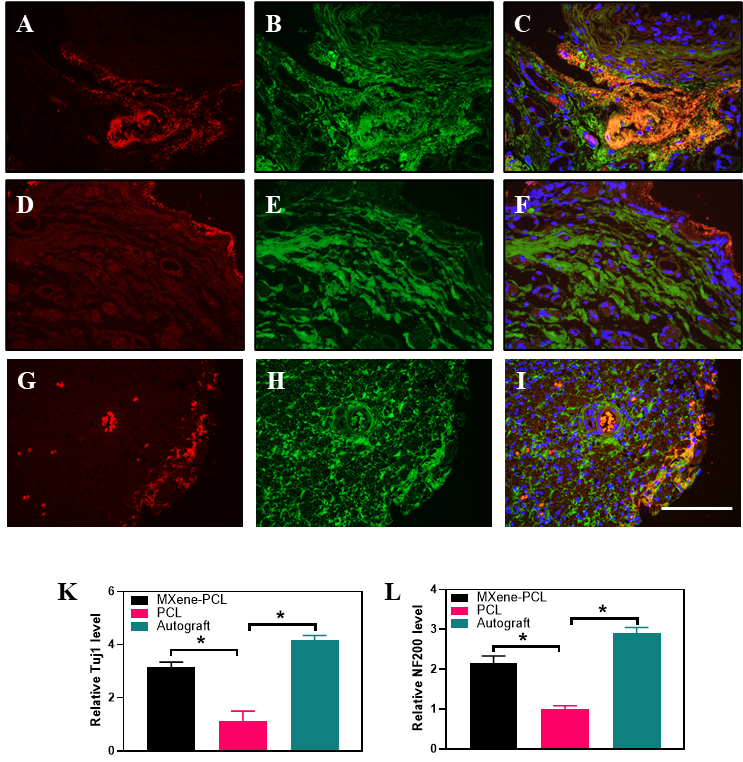


**Fig. S8.** Immunofluorescence staining of Tuj 1 (red) and NF200(green) from MXene-PCL group A-C), PCL group D-F), and autograft group G-I) at 12 weeks postoperatively. Relative expression level of Tuj 1 K) and NF200 L). Experiments were repeated 3 times. **p* < 0.05. The scale bar is 200 μm.
